# Impact of ICU-Based Extracorporeal Membrane Oxygenation and Blood Purification Therapy on the Time Required for Early Rehabilitation

**DOI:** 10.7759/cureus.77685

**Published:** 2025-01-19

**Authors:** Shinichi Watanabe, Tomohiro Yoshikawa, Yoshie Hirota, Yuji Naito, Daisetsu Yasumura, Kota Yamauchi, Keisuke Suzuki, Takayasu Koike, Yasunari Morita

**Affiliations:** 1 Department of Rehabilitation Medicine, Faculty of Rehabilitation, Gifu University of Health Science, Gifu, JPN; 2 Department of Rehabilitation, National Hospital Organization, Nagoya Medical Center, Nagoya, JPN; 3 Department of Rehabilitation Medicine, National Hospital Organization, Hokkaido Medical Center, Hokkaido, JPN; 4 Department of Rehabilitation, National Hospital Organization, Beppu Medical Center, Beppu, JPN; 5 Department of Rehabilitation, National Hospital Organization, Shizuoka Medical Center, Shizuoka, JPN; 6 Department of Rehabilitation, Naha City Hospital, Okinawa, JPN; 7 Department of Rehabilitation, Steel Memorial Yawata Hospital, Kitakyushu, JPN; 8 Department of Physical Therapy, Faculty of Rehabilitation, Gifu University of Health Science, Gifu, JPN; 9 Department of Emergency Medicine, National Hospital Organization, Nagoya Medical Center, Nagoya, JPN

**Keywords:** acute blood purification, early rehabilitation, extracorporeal membrane oxygenation, intensive care unit, mobilization

## Abstract

Background

Early rehabilitation in an intensive care unit (ICU) can shorten the ICU stay and reduce the associated risks and costs. However, mobilization has been shown to be slower and less successful in patients receiving blood purification therapy or extracorporeal membrane oxygenation (ECMO), and the ideal duration of rehabilitation has not been determined. To examine this, the time required for rehabilitation in patients receiving ECMO and blood purification therapy in the ICU was examined.

Materials and methods

This was a post-hoc analysis of the IPAM study (Investigating Physical Activity of Mechanical Ventilation Patients in the ICU). The IPAM study with data from hospitalization between September 2022 and March 2023 is a multicenter observational prospective cohort study. Inclusion criteria were subjects who were supported by mechanical ventilation in the ICU for ≥48 hours. The exclusion criteria were age <18 years, loss of walking independence at least two weeks before admission, neurological complications or lack of communication skills due to preexisting mental conditions, terminal stage, and ongoing coronavirus infection. These cases were divided into an ECMO/blood purification group, in which ECMO and blood purification were performed upon admission to the ICU, and a group that did not receive these interventions. The primary endpoint was the daily duration of rehabilitation activity. The secondary endpoints were the daily durations of preparation and rehabilitation activity and the total ICU stay.

Results

Following case selection, 121 cases were analyzed, 28 in the ECMO/blood purification group and 93 in the control group. Total daily rehabilitation time and ICU stay were significantly longer in the ECMO/blood purification group. Among all cases, eight (1%) adverse events occurred during the 959 rehabilitation interventions. No adverse events such as death, cardiopulmonary resuscitation, falls, or intubation-related problems occurred. Kaplan-Meier curve analysis revealed that sitting (p = 0.042), standing (p = 0.001), and walking (p = 0.001) were significantly delayed in the ECMO/blood purification group.

Conclusion

Patients receiving ECMO/blood purification in the ICU require more rehabilitation activity each day, including longer preparation time.

## Introduction

Although advances in medical technology have significantly improved survival rates for intensive care unit (ICU) patients [1], 40-70% of ICU survivors continue to suffer from functional impairment after ICU discharge [2]. Many of these patients experience long-term impairment in daily activities, and their health-related quality of life continues to decline for years after discharge [3,4]. For patients requiring acute blood purification or extracorporeal membrane oxygenation (ECMO), the deadline for calculating intensive care management fees has been extended from day 14 to day 25 of the ICU stay, based on Japan’s 2022 medical fee revision. These calculation guidelines require that a system to facilitate early recovery must be in place. However, the guidelines do not specify the system required for ICU rehabilitation, and the maximum number of days for rehabilitation remains at 14 [5].

Early rehabilitation in the ICU has attracted international attention as a preventive measure against physical dysfunction in critically ill patients. Early rehabilitation was a set of measures begun within 48 hours for a wide range of patients with a new illness, surgery, or acute exacerbation that helps maintain, improve, or restore a range of functions, including mobility, breathing, feeding/swallowing, and digestion/absorption [6]. Mobilization is an exercise program that includes passive and active exercise, sitting and standing balance training, and gait re-education, with the level of exercise determined by a mobilization protocol and continued until hospital discharge [2,6]. Early rehabilitation can prevent delirium, reduce the period of mechanical ventilation and the ICU stay, improve physical function, and reduce medical costs [2,7,8]. In Japan, owing to the revision of the medical fee system, premiums for early mobilization and rehabilitation were introduced in 2019, and their implementation, mainly by nurses and physical therapists, is increasing [6]. Nonetheless, the ideal duration of rehabilitation has not yet been clearly determined, and facilities are still determining this independently. Patients undergoing ECMO/blood purification have been shown to require a significantly longer time to mobilization and to exhibit significantly lower mean and maximum mobilization levels, than control cases, owing primarily to mobility restrictions caused by the devices used for ECMO and acute blood purification and a lack of staff [9]. For effective rehabilitation, patients who have traditionally been treated via ventilator management or blood purification require longer times for preparation and rehabilitation activity and the cooperation of multiple professionals [9]. Nonetheless, the amount of daily preparation and rehabilitation time required for early rehabilitation in patients undergoing ICU-based ECMO/blood purification remains unknown.

To address this, the time required for preparation and rehabilitation in patients receiving ECMO/blood purification in the ICU was examined. The intensity of the rehabilitation required was compared between the target and control groups. Furthermore, the study examined whether the target group required fewer rehabilitation staff than the control group. The hypothesis of this study was that patients receiving ECMO/blood purification would require more time for preparation and rehabilitation compared to patients not receiving it. The findings of this study suggest ways to improve rehabilitation and mobility in patients receiving ECMO/blood purification in the ICU.

## Materials and methods

Study design and patient selection

This was a post-hoc analysis of the IPAM study (Investigating Physical Activity of Mechanical Ventilation Patients in the ICU) [10]. The IPAM study was approved by the Ethics Committee of Gifu University of Health Science (202204; June 14, 2022) and eight other participating hospitals between April 2019 and March 2020 and was registered with UMIN (ID: 000047578). The IPAM study with data from hospitalization between September 2022 and March 2023 is a multicenter observational prospective cohort study. The IPAM study was conducted according to the STROBE guidelines and informed consent was obtained from all patients. Patients younger than 18 years of age, those with a loss of walking independence before hospitalization, those with neurological complications or a lack of communication skills due to preexisting mental illness, those in a terminal state, and those with did not complete the assessment during ICU stay, and denied consent were excluded. The patients were divided into those who received ECMO or blood purification therapy upon admission to the ICU and a control group of patients admitted to the ICU but who did not receive ECMO/blood purification therapy. Blood purification therapy was defined as the use of all renal support therapies administered during ICU admission, including continuous and intermittent hemodialysis but excluding chronic maintenance dialysis and peritoneal dialysis [11].

Data collection

Patient characteristics included the patient’s age, sex, body mass index (BMI), Charlson index [12], diagnosis at admission, planned postoperative surgery, acute physiology and chronic health evaluation (APACHE II) score, sequential organ failure assessment (SOFA) score, and pre-hospitalization Barthel index.

Primary outcomes

The primary outcome was the duration of daily rehabilitation activity in the ICU. Follow-up continued until the patient left the ICU. Rehabilitation-activity duration was measured in minutes, using a stopwatch, from the start to the end of the intervention (including preparation time and rest time) by the person managing the implementation [10,13].

Secondary outcomes

Secondary outcomes included the duration of daily rehabilitation activity (without preparation time) and that of preparation; days of physical restraint; Medical Research Council (MRC) score at ICU discharge [14]; days of delirium; time to first rehabilitation intervention, first sitting, standing, and walking; length of ICU stay; duration of mechanical ventilation; and adverse events during rehabilitation.

Adverse events that were monitored during rehabilitation included death, cardiopulmonary arrest, inadvertent removal of medical devices, fall to knees or the ground, desaturation (to <80% saturation) or a decline of >10% from baseline saturation, bradypnea (<5 breaths/min) or tachypnea (>40 breaths/min), bradycardia (<40 beats/min) or tachycardia (>130 beats/min), hypotension (systolic blood pressure < 80 mmHg), hypertension (systolic blood pressure > 200 mmHg), and new arrhythmia (defined as arrhythmia lasting longer than a minute). To identify delirium, two screening tools were used: the Confusion Assessment Method for the Intensive Care Unit [15] and the Intensive Care Delirium Screening Checklist [16].

Early rehabilitation protocol

Day 1 was defined as the beginning of the day shift on the first day of admission to the ICU. The protocol began with passive range-of-motion exercises of the extremities while the patient was prone on the bed and gradually transitioned to automatic movement of the extremities. Physical respiratory therapy was administered as needed to improve and prevent pneumonia and atelectasis. As a patient’s general condition stabilizes, they progress to mobilization, sitting, standing, and walking. Decisions on whether to continue or discontinue rehabilitation, which ICU care techniques to implement [17], and whether patients could be moved out of bed each day were taken based on the standards of each facility and in accordance with expert consensus [8] and the PADIS guidelines [18]. The participating facilities extended the overall duration of each rehabilitation session to maintain a constant session duration.

Statistical analysis

The baseline characteristics and clinical outcomes in each group were compared. Differences in continuous variables were evaluated using the Mann-Whitney U test, and differences in nominal variables were performed using chi-squared tests.

To evaluate the impact of blood purification on time to first mobilization, Kaplan-Meier analysis was used to examine the proportions of patients who achieved sitting, standing, and walking before hospital discharge, using log-rank tests for between-group comparisons.

All analyses were performed using JMP version 13.0 (SAS Institute, Cary, NC, USA). Statistical tests were two-sided. Differences were considered significant at p < 0.05.

## Results

Baseline characteristics

**Figure 1:**
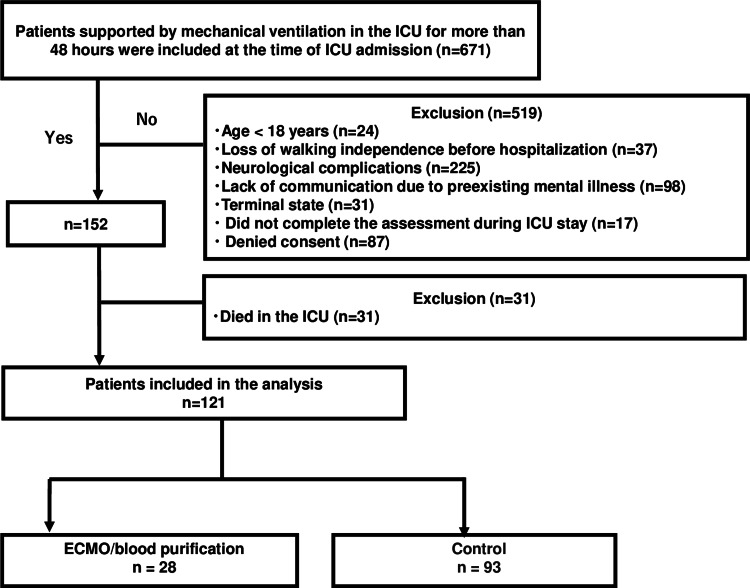
Study flow chart. ICU = intensive care unit, ECMO = extracorporeal membrane oxygenation

Following case selection, 121 patients were included in the final analysis: 28 in the ECMO/blood purification group (five patients on ECMO, 23 on hemodiafiltration, and four on endotoxin adsorption therapy) and 93 in the control group (Figure 1).

At the ICU admission, there were significant differences in APACHE II score, SOFA score, and BMI between the groups (Table 1).

**Table 1:** Baseline characteristics. Data are presented as median [IQR] or n (%) (*p < 0.05). Differences in continuous variables were evaluated using the Mann–Whitney U test (U-value), and differences in nominal variables were performed using chi-squared tests (chi-square value). IQR = interquartile range; ECMO = extracorporeal membrane oxygenation; IQR = interquartile range; BMI = Body mass index; CCI = Charlson comorbidity index; BI = Barthel index; ICU = intensive care unit; APACHE = Acute Physiology and Chronic Health Evaluation; SOFA = Sequential Organ Failure Assessment. ^a ^Breakdown of post-operation: cardiovascular surgery, 31 (51%); gastrointestinal surgery, 20 (33%); other surgery, 10 (16%).

	Total	ECMO/blood purification	Control	Test statistic	p-value
	n= 121	n= 28	n = 93		
Age (years), median [IQR]	74 [64–80]	77 [64–80]	73 [64–80]	1487.0	0.255
Gender (male), n (%)	72 (59)	15 (54)	57 (61)	0.629	0.514
BMI (kg/m2), median [IQR]	21 [18–24]	21 [19–25]	21 [18–24]	1381.5	0.562
Charlson index, median [IQR]	1 [1–3]	1 [1–3]	1 [1–3]	1492.5	0.230
ICU admission diagnosis, n (%)					
Acute respiratory failure	24 (20)	3 (11)	21 (23)	4.112	0.279
Cardiovascular disease	50 (41)	10 (36)	40 (43)		
Gastric or colonic surgery	25 (21)	8 (28)	17 (18)		
Other diagnoses	22 (18)	7 (25)	15 (16)		
Surgical, n (%) ^a^	61 (50)	17 (61)	44 (47)	1.667	0.282
APACHE II score, median [IQR]	22 [16–26]	26 [20–32]	21 [15–25]	1829.2	0.008*
SOFA at ICU admission, median [IQR]	7 [4–10]	10 [6–12]	7 [4–9]	1781.5	0.002*
BI before hospitalization, median [IQR]	100 [100–100]	100 [90–100]	100 [100–100]	948.0	0.002*

Clinical outcomes

The mean daily duration of rehabilitation activity (the primary outcome) was significantly higher in the ECMO/blood purification group than in the control (p = 0.003).

Of the secondary outcomes, relative to the control, the target group exhibited a significantly lower mean duration of daily rehabilitation activity (p = 0.007); higher mean daily rehabilitation-activity frequency (p = 0.034); more days with physical restraint (p=0.016); longer times to first sitting (p = 0.003), standing (p = 0.006), and walking (p = 0.002); longer ICU stay (p = 0.035); longer duration of mechanical ventilation (p = 0.024); higher MRC score at ICU discharge (p < 0.001); and higher BMI at ICU discharge (p = 0.002) (Table 2).

**Table 2:** Comparison of rehabilitation dates and clinical outcomes. Data are presented as median [IQR] (*p < 0.05). Differences in continuous variables were evaluated using the Mann–Whitney U test (U-value). IQR = interquartile range; ICU = intensive care unit; ECMO = extracorporeal membrane oxygenation; MRC = Medial Research Council; BI = Barthel index.

	ECMO/blood purification	Control	Test statistic	p-value
	n= 28	n = 93		
Primary outcomes				
Mean daily rehabilitation time (minute/day)	24.0[17.0–38.2]	16.6 [10.0–22.2]	827.0	0.003*
Secondary outcomes				
Mean daily activity time (minute/day)	12.5 [0–30.5]	20.0 [0–37.5]	1212.0	0.007*
Mean daily rehabilitation frequency (time/day)	1.0 [0.6–1.2]	0.8 [0.3–1.0]	960.0	0.034*
Highest ICU mobility scale	2.5 [0–3.7]	4.0 [3.0–7.0]	697.0	0.408
Rehabilitation daily preparation time (minute/day)	14 [5–21]	6 [0–16]	1136.5	0.016*
Physical restraint days (day)	5.5 [3.0–10.0]	5.0 [3.0–8.0]	1374.0	0.732
prone position therapy (day)	0 [0–0]	0 [0–0]	1281.0	0.016*
Delirium day (day)	0 [0–3]	0 [0–3]	1171.5	0.382
Time to first rehabilitation day (day)	2.3 [0.6–4.6]	1.1 [0.7–2.8]	1527.0	0.167
Time to first sitting day (day)	8.0 [5.3–11.7]	4.8 [2.9–7.4]	1998.0	0.003*
Time to first standing day (day)	8.7 [7.0–16.5]	6.5 [4.6–8.1]	1660.0	0.006*
Time to first walking day (day)	13.7 [10.8–32.9]	8.8 [6.4–15.4]	1484.5	0.002*
Length of ICU stay (day)	9.3 [6.7–11.6]	7.9 [5.0–11.6]	1645.5	0.035*
Duration of mechanical ventilation (day)	6.0 [4.6–17.9]	4.9 [3.0–8.0]	1652.5	0.024*
MRC score at ICU discharge	36 [24–48]	48 [45–56]	559.5	<0.001*
BI at ICU discharge	7.5 [5.0–15.0]	25.0 [5.0–45.0]	797.0	0.002*

Incidence of adverse events

Among all cases, eight (1%) adverse events occurred during the 959 rehabilitation interventions. Among the adverse events, desaturation was the most frequent, with two desaturation events occurring in each group. None of the interventions involved adverse events such as death, cardiopulmonary resuscitation, falls, or intubation-related problems (Table 3).

**Table 3:** Incidence of adverse events. There were 959 rehabilitation sessions in the first 14 days of intensive care unit stay. ECMO = extracorporeal membrane oxygenation.

	ECMO/blood purification (205 sessions)	Event rate per 1000 sessions	Control (754 sessions)	Event rate per 1000 sessions
Death, cardiopulmonary arrest, time	0	0	0	0
Fall to knees or ground, time	0	0	0	0
Inadvertent removal of medical devices, time	0	0	0	0
Desaturation, time	2	10	2	3
Tachypnea or bradypnea, time	0	0	0	1
Tachycardia or bradycardia, time	1	5	1	2
Hypertension or hypotension, time	1	5	1	2
New arrhythmia, time	0	0	0	0

Therapeutic outcomes in rehabilitation

**Figure 2:**
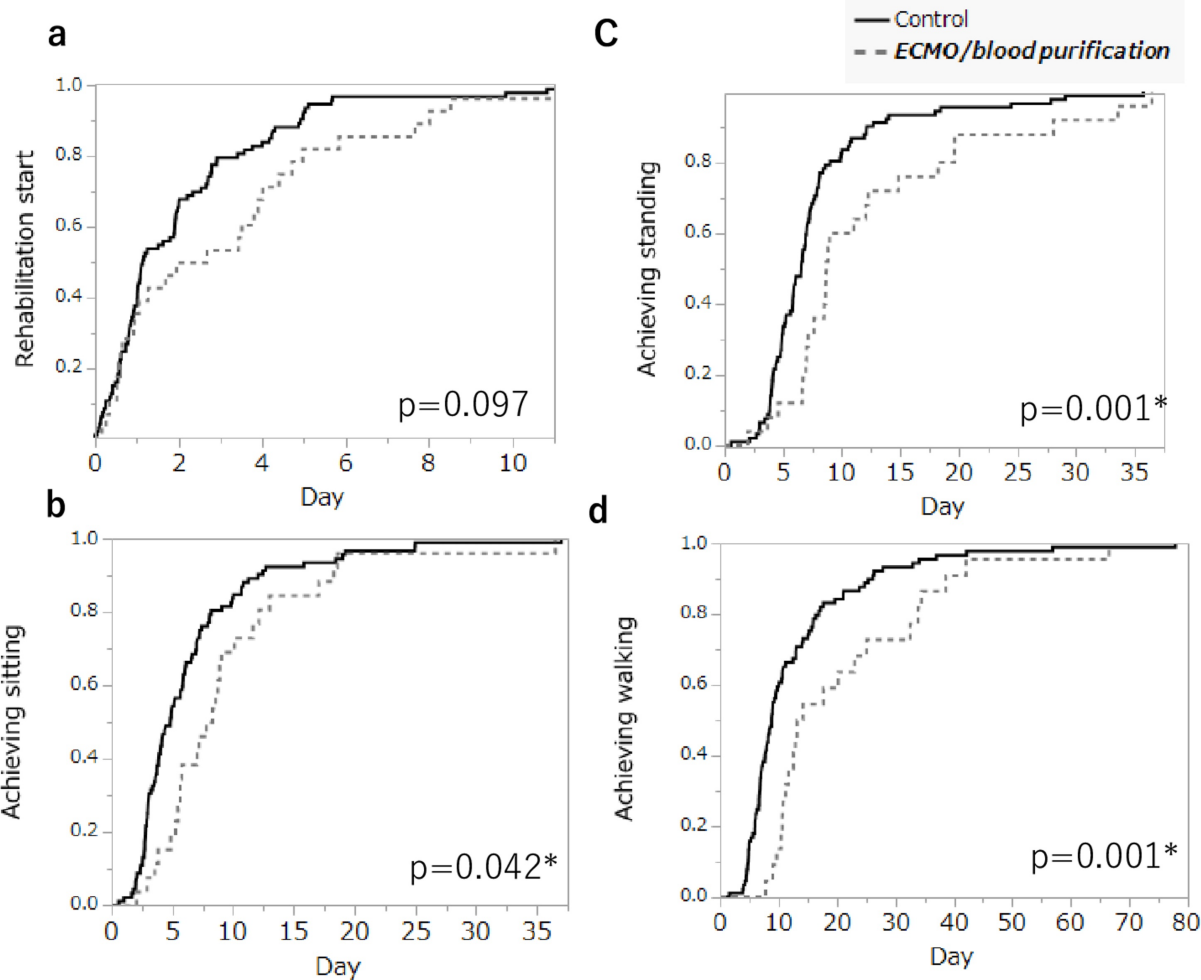
Comparison of therapeutic outcomes in rehabilitation. Data are presented as mean (*p < 0.05). Kaplan-Meier analysis was used, and the log-rank test was used for comparisons between groups. The Kaplan-Meier curves showed that the ECMO/blood purification group had significantly delayed sitting (p = 0.042), standing (p = 0.001), and walking (p = 0.001), whereas no significant difference was observed in the rehabilitation start (p = 0.097). ECMO = extracorporeal membrane oxygenation.

The Kaplan-Meier curve analysis revealed that sitting (p = 0.042), standing (p = 0.001), and walking (p = 0.001) were significantly delayed in the ECMO/blood purification group; at the start of rehabilitation, no significant differences were observed (p = 0.097) (Figure 2).

## Discussion

For clinically ill patients undergoing ECMO/blood purification therapy, barriers to mobilization include circulatory issues and consciousness-related factors such as sedation [9]. However, few studies have examined the required preparation time or whether critically ill patients require longer for rehabilitation. To address this, preparation and implementation times for early rehabilitation in patients receiving ECMO or blood purification therapy were examined. Based on these findings, the total daily duration of rehabilitation activity was significantly higher in the ECMO/blood purification group than in the control.

A few studies have examined the effects of rehabilitation starting time and intensity [7,13]. In a study examining rehabilitation intensity in patients in the ICU, the group that performed high-intensity exercises in the ICU had a significantly higher rate of discharge to home than the low-intensity exercise group [19]. In blood purification therapy, the staff needs to tailor the environment to ensure safe medical care, crisis management, physical restraint to ensure rest, positional management, and rehabilitation in collaboration with various professionals [9]. In this study, the ECMO/blood purification group exhibited more days of physical restraint. In the rehabilitation of patients undergoing ECMO or blood purification therapy, the activity required to prepare for rehabilitation, such as managing the patient’s general condition and adjusting the equipment, is time-consuming. Therefore, to maintain safety, sufficient time should be allocated to prepare for rehabilitation.

Examination of Diagnosis Procedure Combination (DPC) data revealed that patients receiving blood purification therapy exhibited the longest average ICU stay and the highest proportion of ICU stays exceeding 14 days [5]. Similarly, in this study, the group receiving blood purification therapy spent significantly longer in the ICU than the control group. However, under the current medical fee system, at most 14 days are allowed for early bed-based rehabilitation; for patients who stay in ICU for longer than 14 days, this may be insufficient. The Kaplan-Meier results showed a significant delay in sitting, standing, and walking in the ECMO/blood purification group, but no significant differences were observed at the start of rehabilitation. Therefore, the study results should be interpreted with caution.

The early mobilization of ventilated patients carries risks related to the removal of line tubes and adverse circulatory and respiratory reactions. Here, during the 959 rehabilitation sessions, no serious adverse events such as death occurred in either group, and no significant differences were observed between the two groups in terms of vital sign abnormalities. Most of the adverse events occurring during rehabilitation were physiological responses to exercise that can be expected during rehabilitation. These changes are considered clinically acceptable, and recovery from such changes occurs rapidly with rest. In patients undergoing ECMO and blood purification therapy in the ICU, early intervention for rehabilitation can be safely performed when there is multidisciplinary cooperation [20]. The same holds true for patients undergoing blood purification therapy.

This study had several limitations, and the results should therefore be interpreted with caution. This was a secondary analysis of a prospective multicenter study with a limited study period and sample size. The study sample size was limited in this study, and the ECMO/blood purification and control groups were assigned unbalanced. This imbalance reduced the statistical power of this study. As the cases were not randomly assigned to the groups, the results cannot be generalized to patients receiving ECMO/blood purification therapy. Furthermore, although this study evaluated the daily duration of rehabilitation activity, it did not evaluate activities at other times, such as those performed by the patients themselves, and it was not possible to verify the duration of physical activity. Furthermore, in this study, we were unable to collect detailed information about the surgery, such as operation time. In the future, a larger sample should be used and physical activity monitors utilized to verify the results.

## Conclusions

Among these critically ill patients, the total daily duration of rehabilitation activity was significantly higher in the ECMO/blood purification group than in the control. Based on these findings, the early rehabilitation of patients undergoing ICU-based ECMO or blood purification takes longer, requires more preparation time, and requires more multidisciplinary interventions for daily care, than that of other critically ill patients. Further studies are needed to determine whether increasing the amount of rehabilitation in the ECMO/blood purification group will facilitate individuals' return to daily activities.
